# Neuronal Sprouting and Reorganization in Bone Tissue Infiltrated by Human Breast Cancer Cells

**DOI:** 10.3389/fpain.2022.887747

**Published:** 2022-05-31

**Authors:** Rie B. Hansen, Manasi Sayilekshmy, Michala S. Sørensen, Astrid H. Jørgensen, Ida B. Kanneworff, Emma K. E. Bengtsson, Tomas A. Grum-Schwensen, Michael M. Petersen, Charlotte Ejersted, Thomas L. Andersen, Christina M. Andreasen, Anne-Marie Heegaard

**Affiliations:** ^1^Department of Drug Design and Pharmacology, University of Copenhagen, Copenhagen, Denmark; ^2^Musculoskeletal Tumor Section, Department of Orthopedic Surgery, Rigshospitalet, University of Copenhagen, Copenhagen, Denmark; ^3^Clinical Cell Biology Group, Department of Pathology, University of Southern Denmark, Odense, Denmark; ^4^Department of Clinical Research, University of Southern Denmark, Odense, Denmark; ^5^Department of Molecular Medicine, University of Southern Denmark, Odense, Denmark; ^6^Department of Endocrinology, Odense University Hospital, Odense, Denmark

**Keywords:** cancer-induced bone pain, metastatic bone disease, neuronal sprouting, bone, innervation, breast cancer

## Abstract

**Background:**

Pain is a common complication for patients with metastatic bone disease. Animal models suggest that the pain, in part, is driven by pathological sprouting and reorganization of the nerve fibers innervating the bone. Here, we investigate how these findings translate to humans.

**Methods:**

Bone biopsies were collected from healthy volunteers (*n* = 7) and patients with breast cancer and metastatic bone disease (permissions H-15000679, S-20180057 and S-20110112). Cancer-infiltrated biopsies were from patients without recent anticancer treatment (*n* = 10), patients with recent anticancer treatment (*n* = 10), and patients with joint replacement surgery (*n* = 9). Adjacent bone sections were stained for (1) protein gene product 9.5 and CD34, and (2) cytokeratin 7 and 19. Histomorphometry was used to estimate the area of bone marrow and tumor burden. Nerve profiles were counted, and the nerve profile density calculated. The location of each nerve profile within 25 μm of a vascular structure and/or cancer cells was determined.

**Results:**

Cancer-infiltrated bone tissue demonstrated a significantly higher nerve profile density compared to healthy bone tissue. The percentage of nerve profiles found close to vascular structures was significantly lower in cancer-infiltrated bone tissue. No difference was found in the percentage of nerve profiles located close to cancer between the subgroups of cancer-infiltrated bone tissue. Interestingly, no correlation was found between nerve profile density and tumor burden.

**Conclusions:**

Together, the increased nerve profile density and the decreased association of nerve profiles to vasculature strongly suggests that neuronal sprouting and reorganization occurs in human cancer-infiltrated bone tissue.

## Introduction

Cancer patients point to pain as their most important and one of the most distressing symptoms (1). The dissemination of cancer to bone can lead to significant pain, and up to 85% of terminally ill cancer patients suffer from cancer-induced bone pain (2–4).

Mouse models of cancer-induced bone pain have demonstrated substantial sprouting of both sensory and sympathetic fibers giving rise to an increased nerve fiber density as cancer cells invade and proliferate in the bone tissue (5, 6). The nerve sprouting is observed in periosteum, mineralized bone and bone marrow (7, 8). In healthy mouse bone, sensory and sympathetic nerve fibers display a distinct morphology. Sensory fibers present with a long and linear morphology, while sympathetic nerve fibers have a spiral-like appearance and wrap around associated blood vessels (6, 9). Upon the invasion of cancer cells, the morphology changes to a disorganized appearance where sensory and sympathetic nerve fibers are intermingled (6). It has been proposed that such pathological reorganization can render the nerve fibers highly sensitive to movement of the tumor-bearing limb, reflecting movement-evoked pain (10), which is a key symptom of metastatic bone disease (11, 12). Also, formation of neuroma-like structure has been described in animal models of cancer-induced bone pain (6), and it is speculated that these structures may lead to spontaneous discharge contributing to spontaneous breakthrough pain, which has a strong impact on the patients' quality of life (12, 13).

Although the first reports of cancer-induced sprouting in animal models of cancer-induced bone pain occurred more than 15 years ago (8, 14), it is yet unknown how these findings translate to humans with metastatic bone disease. The present study examines the histological appearance of nerve profiles in bone biopsies from breast cancer patients with metastatic bone disease compared to bone biopsies from healthy controls. The density of nerve profiles and their association with vascular structures and cancer cells are evaluated in the bone marrow.

## Materials and Methods

### Human Bone Biopsies

Bone biopsies were collected from healthy volunteers and patients with breast cancer and metastatic bone disease (MBD). The biopsies were either archived transiliac bone samples from the Pathological Biobank at Odense University Hospital obtained as part of a diagnostic procedure (*n* = 20, aged 37–92 years) or collected during joint replacement surgeries at the Department of Orthopedic surgery at Rigshospitalet due to either impending fracture or fracture caused by metastatic burden (MBD surgery, *n* = 9, aged 50–80 years). The diagnostic biopsies were divided into two groups representing biopsies from patients who had not received anticancer treatment within the last two years (MBD untreated, *n* = 10), and biopsies from patients who had received anticancer treatment (chemotherapy, radiotherapy or a combination) within the last year (MBD treated, *n* = 10). None of these patients had received bisphosphonate treatment. Biopsies from joint replacement surgeries were from patients who had not received anticancer treatment three months prior to surgery. Four of these patients had received bisphosphonate treatment. As controls, transiliac biopsies collected from healthy, female volunteers (*n* = 7, aged 25–67 years) were included.

The collection of biopsies was approved by the National Committee on Health Research Ethics (H-15000679, S-20180057, and S-20110112), permission to collect data was granted through local Data Committees (514-0331/19-3000) and the study was conducted in accordance with the Helsinki Declaration. Healthy volunteers and surgery patients gave written informed consent. The National Committee on Health Research Ethics granted permission to obtain archived biopsies without prior written consent.

### Processing Bone Biopsies

Archived and control biopsies were fixed in 4% paraformaldehyde up to 24 h and decalcified in 10% formic acid for about 7 h. Biopsies isolated at joint replacement surgery were fixed at 4°C in 4% paraformaldehyde for 48–120 h and decalcified with 0.4 M EDTA with 0.4% paraformaldehyde at 4°C for 56–71 days. Subsequently, all bone biopsies were dehydrated and embedded in paraffin. Two adjacent 3.5 μm thick sections were cut from each biopsy, mounted on FLEX, IHC Microscope slide (Dako, Agilent, Denmark) and dried overnight at 37°C.

### Immunostaining

The bone sections were deparaffinized, blocked for endogenous peroxidases, subjected to overnight antigen retrieval in Tris-EDTA buffer (pH 9.0) at 60°C, and blocked with 1% casein in Tris-buffered saline to reduce unspecific binding. The first adjacent section was double-immunostained with rabbit anti-protein gene product 9.5 (PGP9.5, sab4503057, Millipore, Denmark), a pan neuronal marker, and mouse anti-CD34 (clone QBEND/10, ab78165, Abcam, Denmark), a vascular marker of endothelial cells. The second section was immunostained for monoclonal mouse anti-cytokeratin 7 and 19 (clone OV-TL 12/30 and A53-B/A2.26, Cell Marque, Denmark) to visualize cancer cells. This section was used as a reference to verify the identification of cancer cells. Primary antibodies were diluted in Renoir Red diluent (PD904, Biocare Medical, Sweden). Primary antibodies were detected with polymeric alkaline phosphatase-conjugated BrightVision poly AB-Anti-Rabbit IgG (Immunologic, VWR, Denmark) or polymeric horseradish-peroxidase-conjugated BrightVision anti-mouse IgG (Immunologic), and visualized with Stay Red (ab103741, Abcam) or Deep Space Black, DSB (bri4015L, Biocare Medical), respectively. All sections were counterstained with Mayer's hematoxylin and mounted with Aquatex (Merck, Denmark). Unspecific staining of secondary antibodies and visualization systems were investigated by the omission of primary or secondary antibodies.

### Histomorphometry

**Figure 1:**
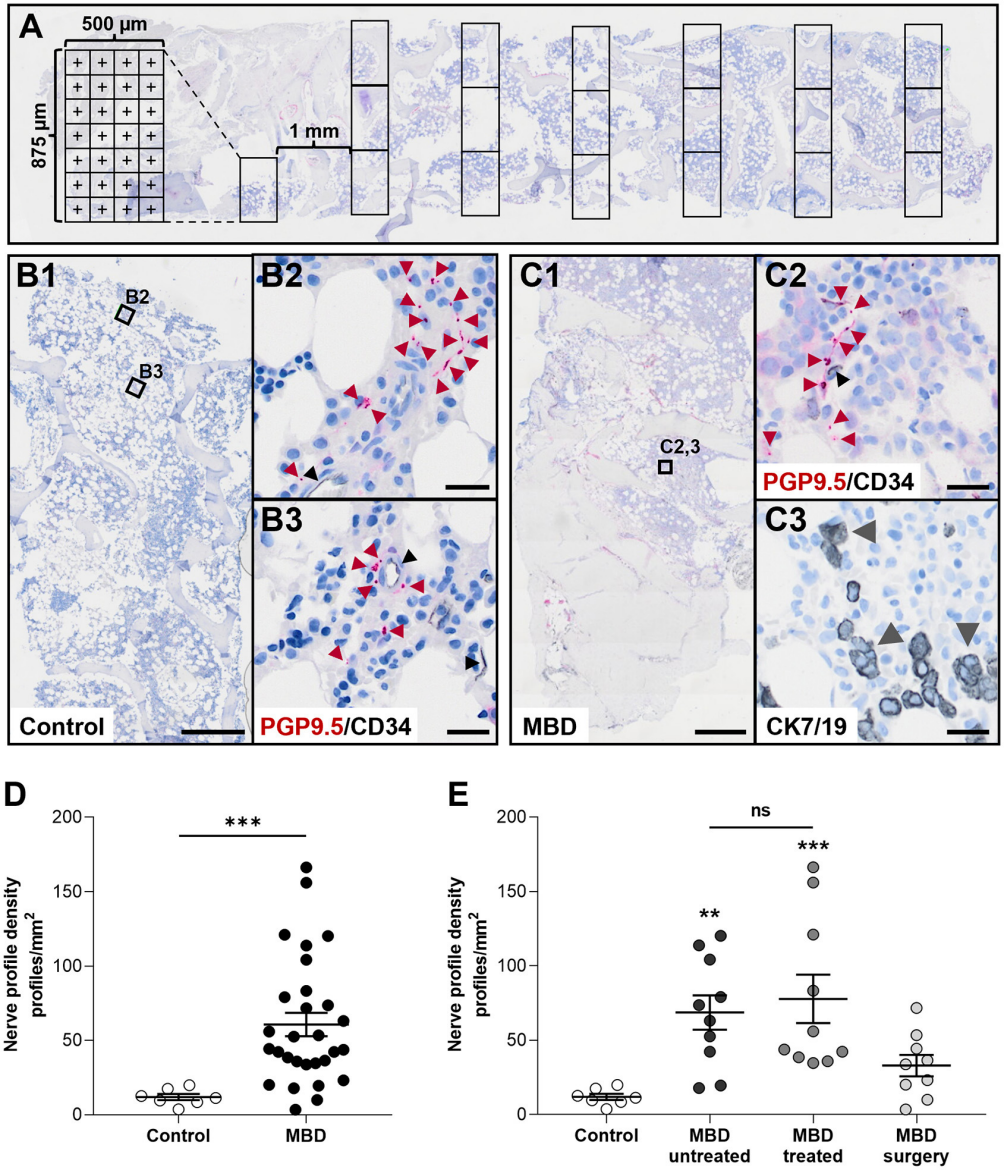
Cancer-infiltrated bone marrow has a significantly increased nerve profile density compared to healthy bone marrow. **(A)** Illustration of the histomorphometric method used to estimate the area of analysis. **(B,C)** Overview of sections **(B1,C1)** and close ups demonstrating the identification of PGP9.5-positive nerve profiles and CD34-positive endothelial cells found in healthy controls **(B2,B3)**, and in the bone marrow of patients with metastatic bone disease (MBD) **(C2)**. For patients with MBD the adjacent section was stained for cytokeratin 7 and 19 (CK7/19, gray arrow heads) to identify cancer cells **(C3)**. **(D)** The nerve profile density was significantly increased in the cancer-infiltrated bone marrow compared to healthy bone marrow. **(E)** Subgroups within the cancer-infiltrated biopsies demonstrated a significant increase in nerve profile density in the bone marrow of both the MBD untreated and MBD treated group compared to healthy bone. No difference was found in between the two groups. Comparisons were performed with Mann-Whitney U test or Kruskal–Wallis one-way analysis of variance followed by Dunn's multiple comparisons test as appropriate. Data are presented as mean ± SEM. **p < 0.01, ***p < 0.001. Scale bars: B1 and C1, 500 μm, B2,3 and C2,3 20 μm.

Histomorphometric analysis was conducted on scans of the immunostained sections. The sections were scanned with a NanoZoomer Slide Scanner (Hamamatsu, Japan), and analyzed using NDP.view 2.8.24 software (Hamamatsu Photonics K.K., EU). In each biopsy, the bone marrow was analyzed using a point-grid to estimate the analyzed area of bone marrow and the area occupied by cancer cells. The point-grid was made on transparent paper with 4 x 7 squares of 125 x 125 μm projected to fit a computer screen with a zoom setting of 10x. The grid was placed in rows across the full width of the biopsy (≥3 mm) at a distance of one mm (twice the height of the grid). If <100 nerve profiles were counted in the analyzed area of a biopsy, additional rows of grids were placed across the biopsy, decreasing the distance of the rows until either >100 nerve profiles were counted or the full area of the bone marrow in the biopsy was analyzed (see Figure 1A for illustration of method). Nerve profiles were identified as individual clear red dots. For each nerve profile it was furthermore determined if it was located within a distance of 25 μm to vasculature and/or cancer cells. Vasculature was identified as CD34-positive structures that presented with a morphology consistent with vasculature. Structures that had a cell-like appearance were excluded as hematopoietic stem cells. The nerve density was estimated as the number of nerves per combined area of bone marrow and cancer cells area (mm^2^). The tumor burden was estimated as the area occupied by cancer cells divided by the combined area of bone marrow and cancer cells. Nerve profiles were counted and marked within the scan, allowing a second experienced observer to review the analysis.

### Statistical Analysis

Statistical analysis was performed with GraphPad Prism (version 9.3.0 GraphPad Software, LLC, CA, USA). D'Agostino-Pearson normality test was used to check if the data was Gaussian distributed. The statistical analysis of differences between nerve profile densities and differences between the percentage of nerve profiles in close association to vasculature or cancer cells was performed with Mann-Whitney U test or Kruskal–Wallis one-way analysis of variance followed by Dunn's multiple comparisons test as appropriate. Spearman correlation was used to assess the linear correlation between nerve profile density and the cancer burden, expressed at the percentage of the total bone marrow that was occupied by cancer cells. For all statistical analyses, a probability value of *p* < 0.05 was considered significant. All data are presented as mean ± standard error of the mean (SEM).

## Results

### Bone Biopsies

The bone biopsies were isolated from healthy female volunteers and patients with metastatic bone disease. Details on patients' age and skeletal site of biopsy isolation are presented in Table 1. The analysis was performed in the bone marrow, in which the cancer-infiltrated biopsies presented with a tumor burden ranging from 0.0 to 71.7%. In the MBD surgery group, 6 out of 9 patients had a fracture. The biopsies varied in overall size and preservation of trabecular bone due to different degrees of cancer-infiltration.

**Table 1:** Patient characteristics.

**Group**	**Control**	**MBD untreated**	**MBD treated**	**MBD surgery**
n	7	10	10	9
Age, median (range)	50 (25–67)	77 (37–87)	62 (50–92)	64 (50–80)
Site of biopsy	Iliac crest	Iliac crest	Iliac crest	Femur, shaft, *n* = 4 Femur, trochanter, *n* = 2 Humerus, shaft, *n* = 2 Pelvis, iliac spine, *n* = 1

### Cancer-Infiltrated Bone Marrow Presents With Enhanced Nerve Profile Density

The nerve profile density was significantly increased in the bone marrow of cancer-infiltrated bone biopsies compared to the bone marrow of healthy bone tissue (60.6 ± 7.8 vs. 11.9 ± 2.1 profiles/mm^2^, *p* < 0.001, Figures 1B–D), ranging from 3.4 to 166.3 profiles/mm^2^ in cancer-infiltrated bone biopsies. Subdivision of the cancer-infiltrated bone biopsies into MBD untreated and MBD treated demonstrated that both groups had a significantly higher abundance of nerve profiles (68.1 ± 11.6 profiles/mm^2^, *p* < 0.01, and 77.8 ± 16.3 profiles/mm^2^, *p* < 0.001, respectively) compared to the control group (11.9 ± 2.1 profiles/mm^2^, Figure 1E). No statistically significant difference in nerve profile density was found comparing the MBD untreated group with the MBD treated group (*p* = 0.8534). In addition, no statistically significant difference was found comparing the MBD surgery group with controls (*p* = 0.2552, Figure 1E).

### Nerve Profiles Are Less Associated With Vasculature in Cancer-Infiltrated Bone Marrow

**Figure 2:**
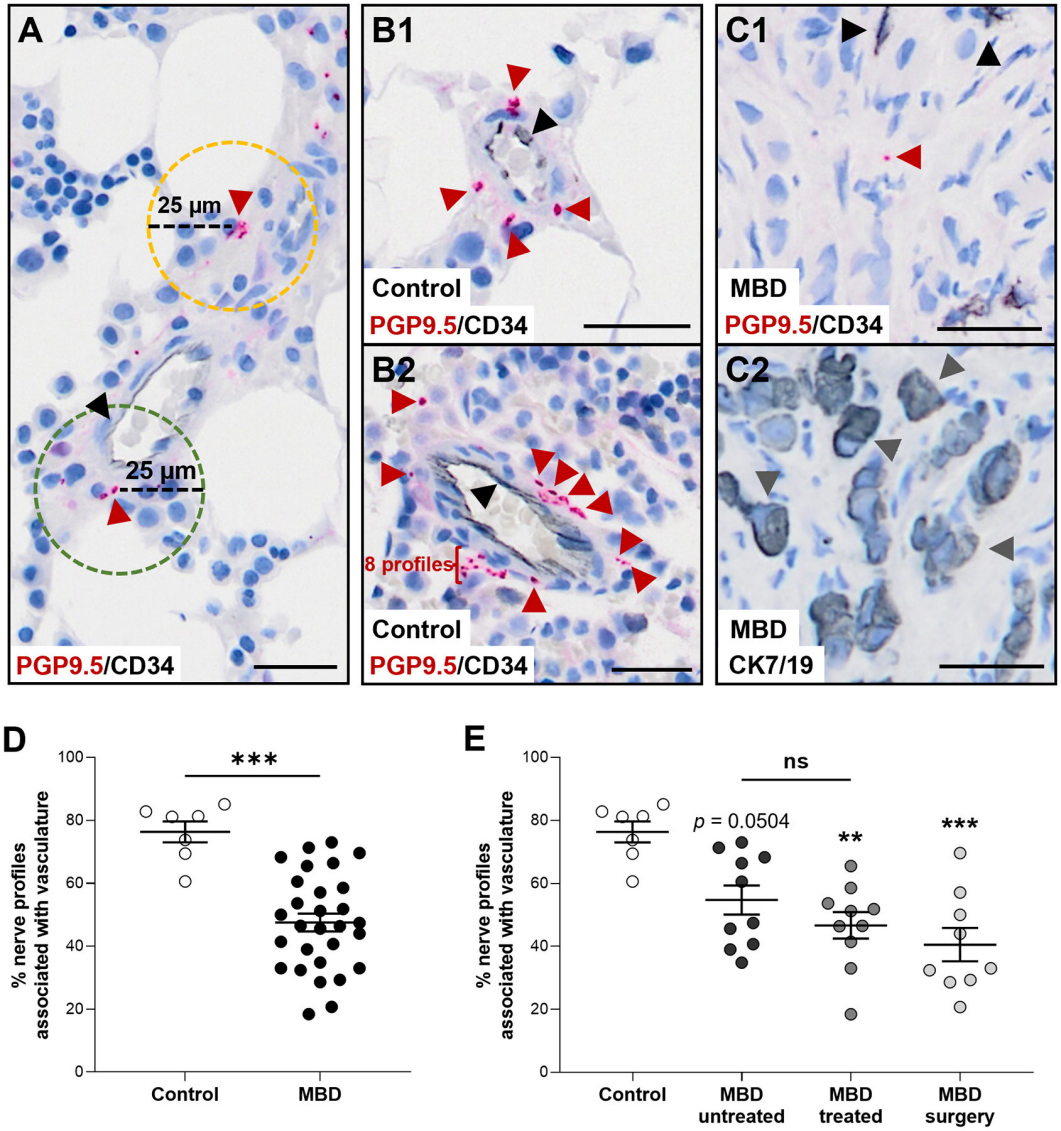
Nerve profiles are less associated with vasculature in cancer-infiltrated bone. **(A)** For each nerve profile it was determined if it was associated within a distance of 25 μm (green hatched line) or unassociated (yellow hatched line) with vasculature. **(B,C)** Representative illustrations of nerve profiles found within 25 μm of a vascular structure in the bone marrow of human healthy tissue **(B1,B2)** and the bone marrow of patients with metastatic bone disease (MBD) **(C1,C2)**. Sections were stained for protein gene product 9.5 (PGP9.5, red arrow heads) and CD34-positive endothelial cells (black arrowheads), while the adjacent section was stained for cytokeratin 7 and 19 [CK7/19, gray arrow heads, **(C2)**] to identify cancer cells. **(D)** The percentage of nerve profiles found proximate to a vascular structure was significantly higher in the healthy bone marrow compared to the cancer-infiltrated bone marrow. **(E)** Analysis of the three subgroups of cancer-infiltrated bone demonstrated a decreased association of nerve profiles and vascular structures in the MBD treated group and the MBD surgery group compared to controls. The comparison of healthy bone tissue with the MBD untreated subgroup did not reach significance (*p* = 0.0504). Comparisons were performed with Mann-Whitney U test or Kruskal–Wallis one-way analysis of variance followed by Dunn's multiple comparisons test as appropriate. Data are presented as mean ± SEM. **p < 0.01, ***p < 0.001. Scale bars: 25 μm.

The association between nerve profiles and vascular structures was determined within the bone marrow. For each nerve profile it was measured whether there was a vascular structure within a 25 μm range (Figures 2A–D). The percentage of nerve profiles near a vascular structure was significantly lower in the bone marrow of cancer-infiltrated bone tissue as compared to healthy bone marrow (47.5 ± 2.8% vs. 76.3 ± 3.3%, *p* < 0.001, Figure 2D). Subdivision of the MBD groups showed that the MBD treated (46.6 ± 4.2%, *p* < 0.01) and MBD surgery group (40.5 ± 5.3%, *p* < 0.001) had a lower percentage of nerve profiles located near vascular structures compared to that of healthy bone tissue (76.3 ± 3.3%, Figure 2E). No difference (*p* = 0.0504) was found between controls and the MBD untreated subgroup (76.3 ± 3.3% vs. 54.7 ± 4.6%, Figure 2E), and no significant difference was found comparing the MBD untreated and MBD treated groups (*p* = 0.3527).

### Nerve Profile Density and Tumor Burden Is Not Correlated in Cancer-Infiltrated Bone Marrow

**Figure 3:**
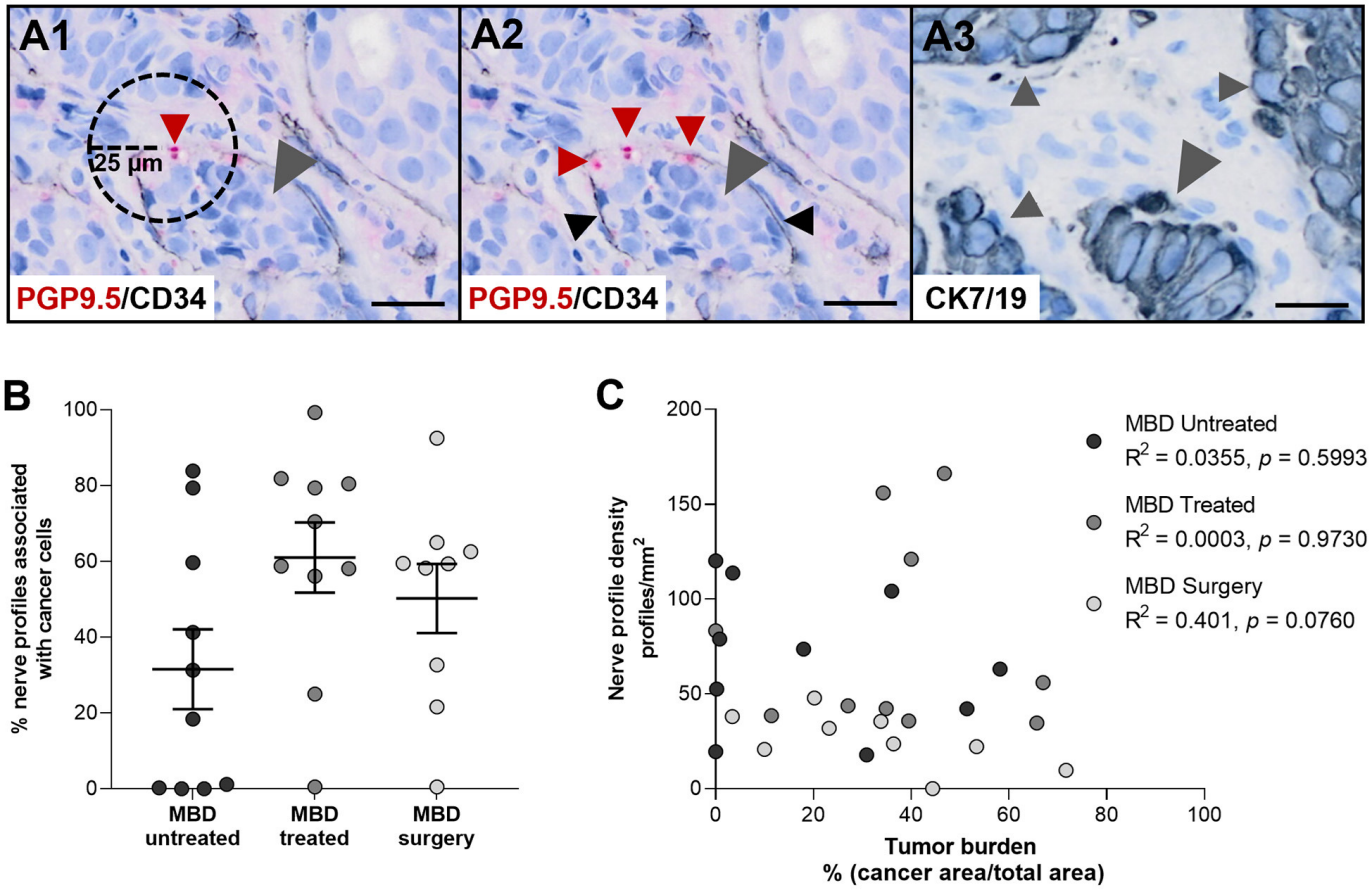
Nerve profile density and tumor burden are not correlated. **(A1)** Illustration of the method. For each nerve profile it was determined if it was located within a distance of 25 μm to cancer cells. **(A2,A3)** Representative illustration of nerve profiles found within 25 μm of cancer cells in the bone marrow of a patient with metastatic bone disease (MBD). Sections were stained for protein gene product 9.5 (PGP9.5, red arrow heads) and CD34 [black arrow heads, **(A2)**], while an adjacent section was stained for cytokeratin 7 and 19 [CK7/19, gray arrow heads, **(A3)**] to identify cancer cells. **(B)** No difference was found in the percentage of nerve profiles found within 25 μm of cancer cells in between the three groups of cancer-infiltrated bone tissue. **(C)** No correlation was found between the nerve profile density and the tumor burden, neither overall nor in any of the subgroups. Analyses were performed with Kruskal–Wallis one-way analysis of variance followed by Dunn's multiple comparisons test and Spearman correlation. Data are presented as mean ± SEM. Scale bars: 25 μm.

To investigate a possible interplay between nerves and cancer cells, the percentage of all identified neuronal profiles located within 25 μm of cancer cells was determined. The mean percentage of neuronal profiles found in close proximity to cancer cells was 31.5 ± 10.5%, 61.0 ± 9.3%, and 50.2 ± 9.1% for the MBD untreated, MBD treated and MDB surgery group, respectively (Figures 3A,B). No difference was found between the three subgroups of cancer-infiltrated bone tissue. Furthermore, the percentages of nerve profiles associated with cancer cells varied greatly with ranges of 0.0–83.9% (MBD untreated), 0.5–99.3% (MBD treated), and 0.5–92.5% (MBD surgery), respectively (Figure 3B). This was in part due to variations in the tumor burden observed in the individual biopsies, which presented with mean and ranges of 19.9 ± 7.2% and 0.0–58.2% (MBD untreated), 36.7 ± 6.6% and 0.0–67.0% (MBD, treated), and 25.5 ± 4.9% and 3.4–71.7% (MBD surgery), respectively. A correlation of nerve profile density to tumor burden demonstrated no apparent relationship, neither overall nor in either of the subgroups (overall correlation, *R*^2^ = 0.0309, *p* = 0.3613, Figure 3C). Hence, there is no indication that nerve sprouting mostly occurs near cancer cells.

## Discussion

Here we provide first evidence that neuronal sprouting and reorganization occur in human breast-cancer infiltrated bone tissue. Firstly, we observe a significant increase in the nerve profile density of cancer-infiltrated human bone marrow tissue, and secondly, we find that these nerve profiles are less associated with vascular structures.

Overall, we found a five-fold increase in the nerve profile density of cancer-infiltrated human bone marrow compared to the healthy control bone marrow. Of note, the level of nerve profile density in the control biopsies was comparable to what was previously described in the bone marrow of patients with primary hyperparathyroidism (15). A recent study, investigating innervation in axial and appendicular human cadaveric bone, demonstrated that the numbers of nerve fibers significantly declined per year of age in elderly individuals (age range 66–99). Extrapolating this finding to younger individuals, it is possible that the increase in nerve profile density in MBD is even higher in magnitude due to the lower median age of the healthy controls (16). The subgroup analysis revealed a significant increase in the nerve profile density in both groups of archived cancer-infiltrated bone marrow. Interestingly, anti-cancer treatment within two years of obtaining the bone marrow biopsy did not seem to affect the nerve density. No significant change in nerve profile density was identified in the cancer-infiltrated biopsies collected during joint replacement surgeries. These biopsies were isolated from various locations in femoral, humeral, and pelvic bones and thus represent a more heterogeneous composition than the biopsies isolated from the iliac crest. Additionally, these biopsies were decalcified in EDTA for 56–71 days, and although EDTA in general is believed to preserve epitopes, the specific effect on PGP9.5 is unknown. That said, in mouse models of cancer-induced bone pain, the bone marrow space has been described to become devoid of nerve fibers as it gets filled with tumor (8), and it is tempting to speculate that the non-significant increase in nerve profile density seen in bone biopsies from patients undergoing joint replacement surgery is due to a beginning denervation. In support, we do not observe any nerve profiles in necrotic areas, visually observed as areas without apparent cell nuclei. In addition, six of nine biopsies were isolated from the intramedullary space of the femoral or humeral shaft, which is the area where cancer cells are seeded in the mouse models of cancer-induced bone pain.

Pathological sprouting of nerve fibers is not only described in models of cancer-induced bone pain, but also in the periosteum of geriatric mice with painful arthritic joints and in painful non-healed bone fractures (17, 18). In the model of cancer-induced bone pain, nerve sprouting is in part believed to drive the pain, as blockade of the nerve sprouting can attenuate the development of late-stage pain related behaviors (6, 19). Nerve growth factor (NGF), released by cancer cells and associated stromal cells, is thought to be a key player in the nerve sprouting as therapies that block the NGF/tropomyosin receptor kinase A (TrkA) pathway in early stages block the sprouting and effectively attenuate the development of cancer-induced bone pain (6, 19). Interestingly, results from a placebo-controlled proof-of concept study and a non-controlled open-label study with tanezumab, a humanized monoclonal antibody that blocks the binding of NGF to TrkA, suggest that the NGF-driven pathology also applies to patients with metastatic bone disease (20). In this study, we did not have access to pain reports from patients; therefore it was not possible to investigate a potential relationship between bone pain and the increase in nerve profile density. However, the significant increase in nerve profile density found in our study combined with the effect of tanezumab in clinical studies suggest that the same mechanism may in part drive the pain in human metastatic bone disease.

Myelinated and unmyelinated fibers have been demonstrated to be mostly associated with vascular structures in human bones (15, 16, 21). In a recent study, examining the association of nerve profiles to vasculature in the bone marrow of iliac crest biopsies isolated from patients with primary hyperparathyroidism, we found that more than 90% of the nerve profiles were associated with vasculature (15). This is in accordance with the present finding in the control biopsies where the majority of nerve profiles were associated with vasculature. In the bone marrow of cancer-infiltrated bone tissue, we found a significantly lower percentage of nerve profiles associated with vasculature compared to healthy bone marrow tissue. This may indicate that structural changes occur in the neuronal network leading to a disorganization of the nerves. Of note, the association of nerve profiles to vascular structures was investigated from the innervation perspective only. Thus, the present data do not provide information on specific types of vasculature or any information on the total coverage of vascular structures. As a qualitative observation, the vasculature of the cancer-infiltrated bone marrow appeared denser and more disorganized as compared to the vasculature of the healthy bone tissue (not shown). Following inoculation of prostate cancer cells, the vasculature of mouse femurs has been reported irregular and disorganized (5). In human breast cancer, hyper-vascularization is generally observed and has been shown to play a role in cancer development, invasion, and metastasis (22). The increased and irregular vascular network visually observed in this study could indicate that cancer cells have the same effect on the vasculature in human bone.

We did not identify any correlation between tumor burden and nerve profile density suggesting that the sprouting is independent of close contact to cancer cells, but instead may be driven by humoral factors. A few cancer-infiltrated bone biopsies would present with no or very few cancer cells. Due to the method of area estimation, the latter would end up with a tumor burden of 0.0%. However, in these biopsies an increased density of neuronal profiles ranging from 19.5 to 120.2 profiles/mm^2^ was still evident_._ Of note, since the analysis is limited to a thin, 2-dimensional section, it is only possible to identify the 2-dimensional nerve profiles and their in-plane association with vasculature and cancer cells. In reality, nerves are elongated 3-dimensional structures that could be associated with vasculature and cancer cells above or below the section plane, meaning that the reported percentages underestimate the 3-dimensional associations with vasculature and cancer cells. In addition, the bone biopsies were isolated from different skeletal sites. Most biopsies were from the iliac crest, but the biopsies from the joint replacement surgeries came from femoral, humeral, and pelvic bones. It has recently been demonstrated that there are differences within the nerve profile density of different bone compartments, i.e. periosteum, cortical pores and bone marrow (15), but it is yet unknown if there are differences between individual skeletal sites, including the weight bearing long bones and less strained bones, such as the iliac crest. Lastly, it is not possible to determine how progressed the cancer is, as patients with metastatic disease per definition are in the most advanced stage of breast cancer (stage IV).

In conclusion, the increased nerve profile density and decreased association of nerve profiles to vasculature found in cancer-infiltrated biopsies strongly suggest that neuronal sprouting and reorganization occur in human cancer-infiltrated bone tissue.

## Data Availability Statement

The raw data supporting the conclusions of this article will be made available by the authors, without undue reservation.

## Ethics Statement

The studies involving human participants were reviewed and approved by National Committee on Health Research Ethics, Denmark, and The Regional Committee, The Capital Region of Denmark. The patients/participants provided their written informed consent to participate in this study. The National Committee on Health Research Ethics granted permission to obtain archived biopsies without prior written consent.

## Author Contributions

MSø, TG-S, and MP enrolled patients and obtained surgical biopsies. Archived samples from Pathological Biobank, OUH were collected by EB and CA. Bone biopsies from healthy controls were collected by CE and TA. RH and CA designed the experiments. RH, AJ, and IK acquired and analyzed the data. EB and MSa acquired data. RH, A-MH, and CA wrote the manuscript. A-MH supervised the study. All authors contributed to the article and approved the final version.

## Funding

The study was supported by the Danish Private Funding Body Advokat Bent Thorbergs Fond and by European Union's Horizon 2020 Research and Innovation Programme under the Marie Sklodowska-Curie grant agreement No. 642720.

## Conflict of Interest

The authors declare that the research was conducted in the absence of any commercial or financial relationships that could be construed as a potential conflict of interest.

## Publisher's Note

All claims expressed in this article are solely those of the authors and do not necessarily represent those of their affiliated organizations, or those of the publisher, the editors and the reviewers. Any product that may be evaluated in this article, or claim that may be made by its manufacturer, is not guaranteed or endorsed by the publisher.
